# Enriched Graphene Oxide-Polypropylene Suture Threads Buttons Modulate the Inflammatory Pathway Induced by *Escherichia coli* Lipopolysaccharide

**DOI:** 10.3390/ijms24076622

**Published:** 2023-04-01

**Authors:** Luigia Fonticoli, Francesca Diomede, Antonio Nanci, Antonella Fontana, Ylenia Della Rocca, Dainelys Guadarrama Bello, Serena Pilato, Oriana Trubiani, Jacopo Pizzicannella, Guya Diletta Marconi

**Affiliations:** 1 Department of Innovative Technologies in Medicine & Dentistry, University “G. d’Annunzio” Chieti-Pescara, Via dei Vestini, 31, 66100 Chieti, Italy; 2 UdA TechLab, University “G. d’Annunzio” Chieti-Pescara, 66100 Chieti, Italy; 3 Laboratory for the Study of Calcified Tissues and Biomaterials, Department of Stomatology, Faculty of Dental Medicine, Université de Montréal, Montreal, QC H3C3J7, Canada; 4 Department of Biochemistry and Molecular Medicine, Faculty of Medicine, Université de Montréal, Montreal, QC H3C3J7, Canada; 5 Department of Pharmacy, University “G. d’Annunzio” Chieti-Pescara, Via dei Vestini, 31, 66100 Chieti, Italy; 6 Department of Engineering and Geology, University “G. d’ Annunzio” Chieti-Pescara, Viale Pindaro, 42, 65127 Pescara, Italy

**Keywords:** graphene oxide, inflammation, suture threads, inflammasome, polypropylene-GO composite

## Abstract

Graphene oxide (GO), derived from graphene, has remarkable chemical–physical properties such as stability, strength, and thermal or electric conductivity and additionally shows antibacterial and anti-inflammatory properties. The present study aimed to evaluate the anti-inflammatory effects of polypropylene suture threads buttons (PPSTBs), enriched with two different concentrations of GO, in the modulation of the inflammatory pathway TLR4/MyD 88/NFκB p65/NLRP3 induced by the *Escherichia coli* (*E. coli*) lipopolysaccharide (LPS-E). The gene and the protein expression of inflammatory markers were evaluated in an in vitro model of primary human gingival fibroblasts (hGFs) by real-time PCR, western blotting, and immunofluorescence analysis. Both GO concentrations used in the polypropylene suture threads buttons-GO constructs (PPSTBs-GO) decreased the expression of inflammatory markers in hGFs treated with LPS-E. The hGFs morphology and adhesion on the PPSTBs-GO constructs were also visualized by inverted light microscopy, scanning electron microscopy (SEM), and real-time PCR. Together, these results suggest that enriched PPSTBs-GO modulates the inflammatory process through TLR4/MyD 88/NFκB p65/NLRP3 pathway.

## 1. Introduction

Graphene, a two-dimensional (2D) nano-structure containing *sp*^2^ carbon atoms, is a building block of several carbon-based materials, including graphite, bucky balls, and carbon nanotubes [1,2]. Graphene was discovered in 2004, and it appeared as a promising nanomaterial due to its catalytic, optical, and electrical properties as well as remarkable physical properties such as a large specific surface area and mechanical strength [3]. In the medical and biological fields, the usefulness of graphene and its derivatives is due to their ability to improve the biocompatibility of various materials already used in tissue engineering [4]. For example, the high aspect ratio, planar structure, flexibility, and hybridization of carbon atoms of graphene help to increase some material properties such as stability [5], strength [6,7], and electric conductivity [8,9]. Graphene oxide (GO) is a nanomaterial derived from the oxidation of graphene, already used in countless electronic, environmental, medical, and biological applications [10,11]. GO is a layer of carbon atoms organized to form a series of hexagons, and unlike graphene, it has functional groups such as hydroxyl (–OH) and epoxy (C-O-C) groups bonded to the underlying graphene plane, while the edges of the sheet are functionalized with carboxylic groups (–COOH) [12,13]. Due to the presence of these chemical groups, GO keeps some properties typical of graphene, such as strength, high mechanical stiffness, transparency, and flexibility [14]. Moreover, it can be easily dispersed in water, and its functional groups allow easy further functionalization or grafting. Studies have proven that graphene and GO are highly biocompatible with low toxicity levels [15] and excellent cytocompatibility [16], which enhance their use as a support for tissue regeneration, cell growth, and cell differentiation [17], at least for the concentration of 10 μg/mL or lower [18]. The innumerable properties of graphene and its derivatives have prompted biomedical research to evaluate the possible application of these materials in the medical field and to study their interaction with the biological system [19]. Over the years, several studies have highlighted how these nanomaterials play a key role in the modulation of biological processes such as inflammation and apoptosis [20], and it was also evidenced that they promote cell adhesion, cell growth, and antibacterial activity [21]. The antibacterial activity of GO includes different mechanisms, such as membrane damage due to sharp edges and oxidative stress [22,23] and bacterial cell wrapping [24]. The biological effect of GO is still not well understood, and different mechanisms have been highlighted depending on the physico-chemical features of GO, its functionalization and dimensions or the tested material in which it is embedded, and the type of investigated cells [25]. The mechanism of cell GO interactions ranges from masking, piercing, rippling, pore formation (generally via membrane lipid extraction), electron transfer, and cation chelation, and it may or not provide internalization into cells. Moreover, differences in cell culture conditions may come into play [26].

The wide increase of studies regarding the properties of GO is strongly encouraged by the fact that graphene is renewable as it can be easily obtained by renewable sources such as lignin [27], is cheap, easily functionalized, and is a one-atom-thick molecule. It is effective at very low concentrations.

The innumerable properties of the GO make its association with various biomaterials an interesting approach in the tissue engineering field. To date, different materials, including many types of suture threads, such as polyglycolic acid (PGA) multifilament surgical and chitin monofilament absorbable surgical sutures, have been functionalized with GO to improve the suture surface wettability and its tensile strength [28,29]. Among the materials used in the production of sutures, polypropylene (PP) is one of the most commonly used. The PP suture is generally a non-absorbable monofilament formed by the catalytic polymerization of propylene. This polymer gives the suture long-term tensile strength superior to that of other materials, such as nylon. For this reason, it is safe in many applications, such as general surgery, as well as in procedures of vascular and cardiac surgery [30].

In the present work, polypropylene suture thread buttons (PPSTBs) obtained from the same material used to produce polypropylene suture threads have been utilized for easy handling. In detail, PPSTBs have been obtained from Assut Europe S.p.A company (Magliano De Marsi (AQ), Italy) by mixing PP enriched with GO at two different concentrations.

Lipopolysaccharide (LPS) is present in the membrane of Gram-negative bacteria, such as *E. coli*, and is also called endotoxin. Bacterial endotoxins are involved in the pathogenesis of Gram-negative sepsis. Infections following injuries, burns, or surgery can lead to the growth of bacterial endotoxins, such as those produced by *E. coli* in the bloodstream [31,32].

The endotoxin of *E. coli*, in contact with the cells, causes the release of pro-inflammatory cytokines after the activation of the Toll-like receptor (TLR) 2 and TLR4, thus stimulating an immune–inflammatory response [33,34]. The major part of Gram-negative bacteria is recognized to induce the production of pro-inflammatory cytokines principally through TLR4 and nuclear factor-κB (NF-κB) pathways [35]. As reported by Pansani T.N. et al., the TLR4 pathway activation is involved in the production of pro-inflammatory cytokines as interleukin-6 (IL-6) and interleukine-8 (IL-8), which was also observed in gingival fibroblasts stimulated with *E. coli* LPS (LPS-E) [36]. Moreover, LPS-E induced a higher expression of inducible nitric oxide (iNOS), IL-6, and monocyte chemotactic protein-1 (MCP-1) in an in vitro model of gingival fibroblast cells stimulated with *E. coli* rather than gingival fibroblasts stimulated with *P. gingivalis* LPS [34].

Based on this knowledge, the purpose of the current work was to analyze the biological effects of PPSTBs enriched with two different concentrations of GO in an in vitro model of primary human gingival fibroblasts (hGFs) to evaluate the potential protective role of PPSTBs functionalized with GO in the inflammatory process through modulation of the TLR4/MyD88/NFκB p65/NLRP3 pathway.

## 2. Results

### 2.1. PPSTBs, PPSTBs-GO 5 μg/mL, and PPSTBs-GO 10 μg/mL Characterization

**Figure 1:**
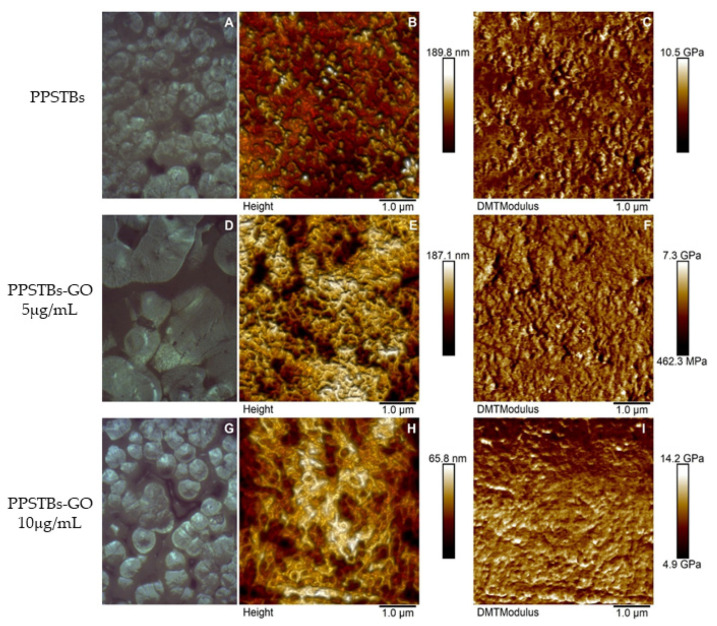
Optical images, AFM micrographs of 2D surface topography, and DMT Modulus channels of PPSTBs (**A**–**C**), PPSTBs-GO 5 μg/mL (**D**–**F**), and PPSTBs-GO 10 μg/mL (**G**–**I**) samples.

In Figure 1, AFM topographical micrographs as well as DMT modulus channels of bare and GO-enriched PPSTBs composites, were reported. By using the Peak Force QNM mode, Young’s elastic modulus for the three samples was obtained. Mean values of Young’s modulus of 4.22 ± 1.49 GPa and 4.39 ± 0.99 GPa were recorded for the PPSTBs and PPSTBs-GO 5 μg/mL samples, respectively, whereas Young’s elastic modulus of 8.40 ± 1.39 GPa was obtained for PPSTBs-GO 10 μg/mL samples.

The diffraction pattern of PPSTBs and PPSTBs-GO, reported in Figure S3 in the Supplementary Materials, showed typical diffraction peaks of PP in the 2*ϴ* range comprised between 10° and 30°. These peaks were related to the crystalline phase of the isotactic PP (i-PP) located at 14°, 17°, 18.5°, 21°, and 22° corresponding to the indexed planes of the monoclinic crystals of the α-form of i-PP (110), (040), (130), (111), and (131) + (041), and to the trigonal crystals of the β-form at 16° and 21° corresponding to the indexed reflections of (300) and (301), respectively [37]. The absence of peaks connected to GO ((001) peak that typically appears between 9–12° 2*ϴ*) in the diffraction patterns of PPSTBs-GO 5 μg/mL and PPSTBs-GO 10 μg/mL indicated that the nanocomposites did not possess layered GO. The addition of GO, even at the highest investigated concentration, did not significantly alter the diffraction pattern of PP. The only difference in the diffraction pattern upon enrichment with GO was the disappearance of the β phase, likely due to a different cooling rate in the crystallization region or nucleation of β crystallites [38].

### 2.2. Cell Viability Assay

**Figure 2:**
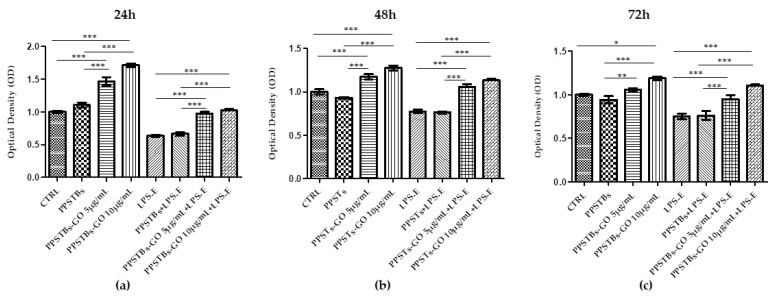
The cell metabolic activity of hGFs with PPSTBs enriched with GO 5 μg/mL and GO 10 μg/mL at (**a**) 24 h, (**b**) 48 h, and (**c**) 72 h. * *p* < 0.05; ** *p* < 0.01; *** *p* < 0.001.

MTS assay was performed on hGFs, hGFs + PPSTBs, hGFs + PPSTBs-GO 5 μg/mL, and hGFs + PPSTBs-GO 10 μg/mL cultured with or without LPS-E at 24, 48, and 72 h (Figure 2). Cell viability was significantly increased in the samples with PPSTBs-GO 5 μg/mL and PPSTBs-GO 10 μg/mL compared to PPSTBs and CTRL samples. The cell metabolic activity increased in hGFs with PPSTBs functionalized with GO with or without the LPS-E treatment.

### 2.3. hGFs Morphological Analysis

**Figure 3:**
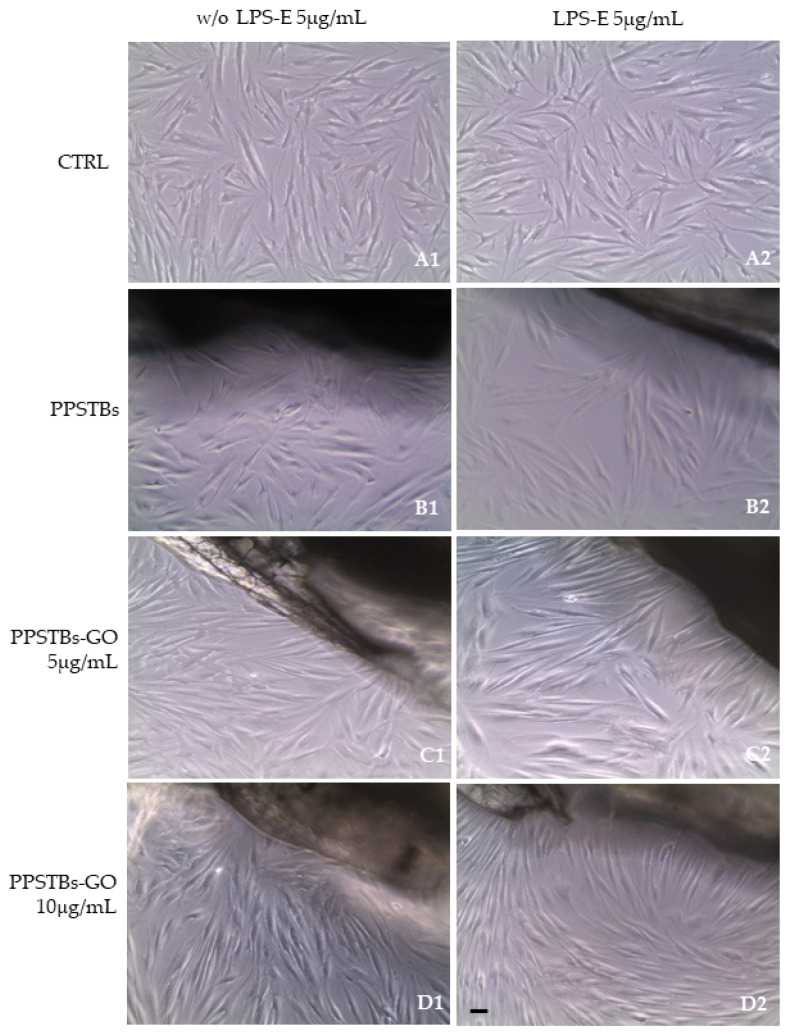
(**A1**–**D1**) The morphology of hGFs alone or cultured with PPSTBs, PPSTBs-GO 5 μg/mL, and PPSTBs-GO 10 μg/mL were observed at the inverted light microscope. (**A2**–**D2**) The morphology of hGFs alone or cultured with PPSTBs, PPSTBs-GO 5 μg/mL, and PPSTBs-GO 10 μg/mL and induced with LPS-E were observed at the inverted light microscope. Scale bar: 20 μm.

After 24 h of LPS-E treatment, the morphology of hGFs alone or cultured with PPSTBs, PPSTBs-GO 5 μg/mL, and PPSTBs-GO 10 μg/mL were observed using an inverted light microscope and SEM. No morphological differences have been observed among all the experimental conditions at the inverted light microscope (Figure 3A1–D2).

**Figure 4:**
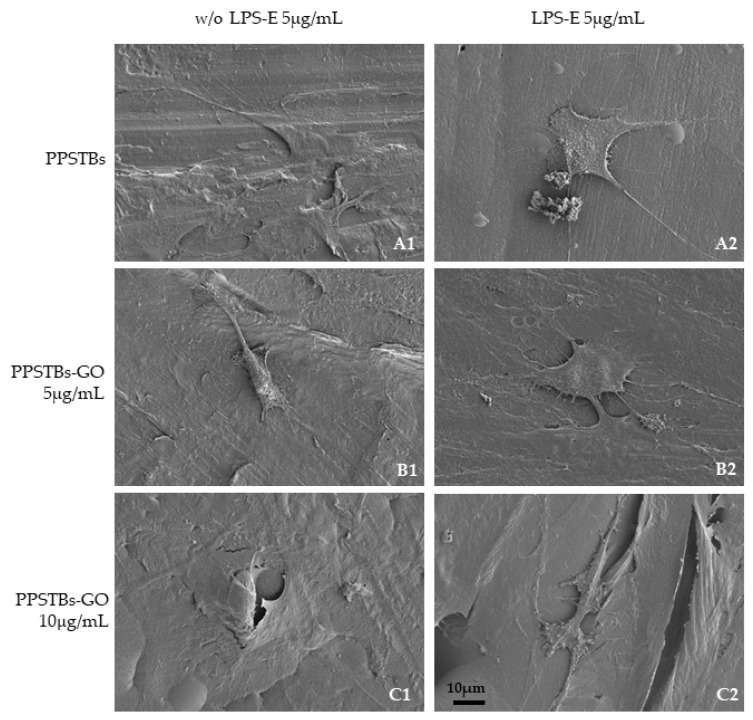
(**A1**–**C1**) Representative SEM images of hGFs, cultured for 24 h on PPSTBs, PPSTBs-GO 5 μg/mL, and PPSTBs-GO 10 μg/mL in the absence of LPS-E treatment. (**A2**–**C2**) hGFs, cultured for 24 h on PPSTBs, PPSTBs-GO 5 μg/mL, and PPSTBs-GO 10 μg/mL with LPS-E. hGFs developed abundant filopodia favoring attachment to the surface and revealing that cells grow in the presence of LPS-E and with different concentrations of GO. Scale bar 10 μm.

The SEM images showed that hGFs adhere equally on PPSTBs, PPSTBs-GO 5 μg/mL, and PPSTBs-GO 10 μg/mL both in the presence or in the absence of LPS-E treatment (Figure 4).

### 2.4. GO-Enriched PPSTBs Influence Protein Expression Evidenced by CLSM and Western Blot Analyses

**Figure 5:**
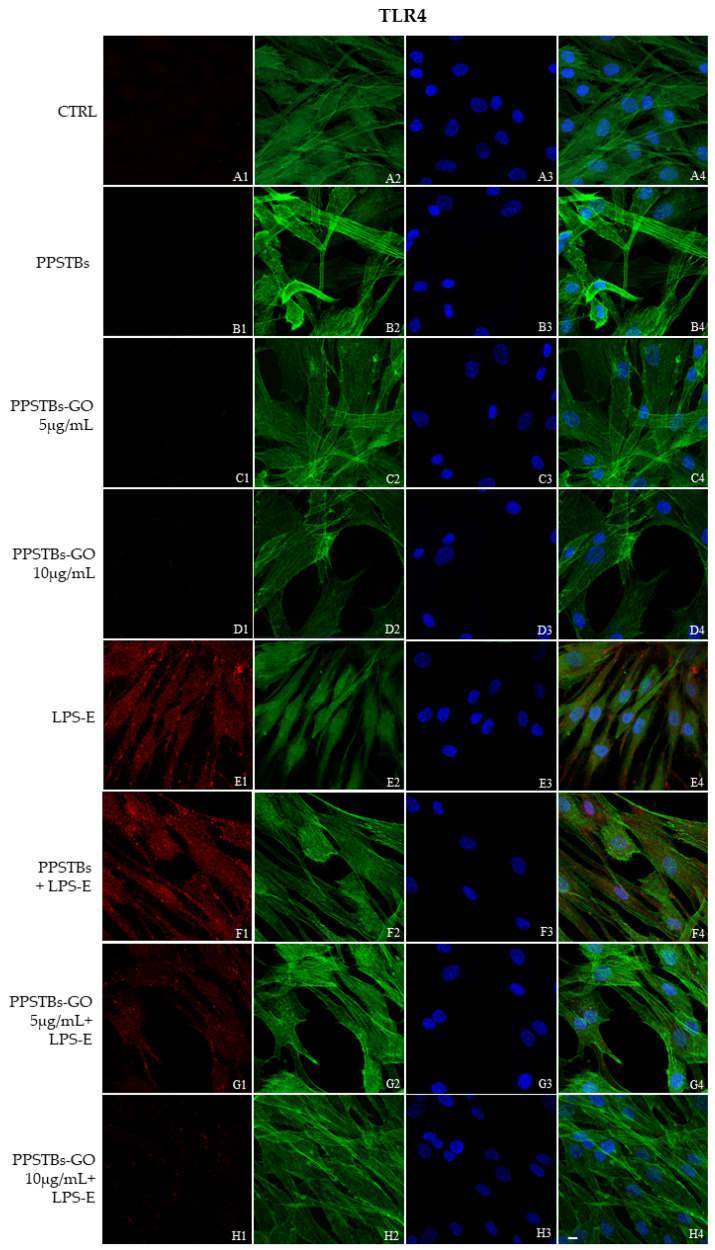
TLR4 signaling pathway in hGFs cell line. Expression of TLR4 analyzed by confocal microscopy (**A1**–**H4**), TLR4 expression in untreated cells (CTRL), in hGFs cultured with PPSTBs, in hGFs cultured with PPSTBs enriched with GO at 5 μg/mL, in hGFs cultured with PPSTBs enriched with GO at 10 μg/mL, in hGFs stimulated with LPS-E, in hGFs cultured with PPSTBs and stimulated with LPS-E, in hGFs cultured with PPSTBs enriched with GO at 5 μg/mL and stimulated with LPS-E and in hGFs cultured with PPSTBs enriched with GO at 10 μg/mL and stimulated with LPS-E. Red fluorescence: TLR4 (**A1**–**H1**); Green fluorescence: cytoskeleton actin (**A2**–**H2**); Blue fluorescence: cell nuclei (**A3**–**H3**) and Merge (**A4**–**H4**). Scale bar: 20 μm.

**Figure 6:**
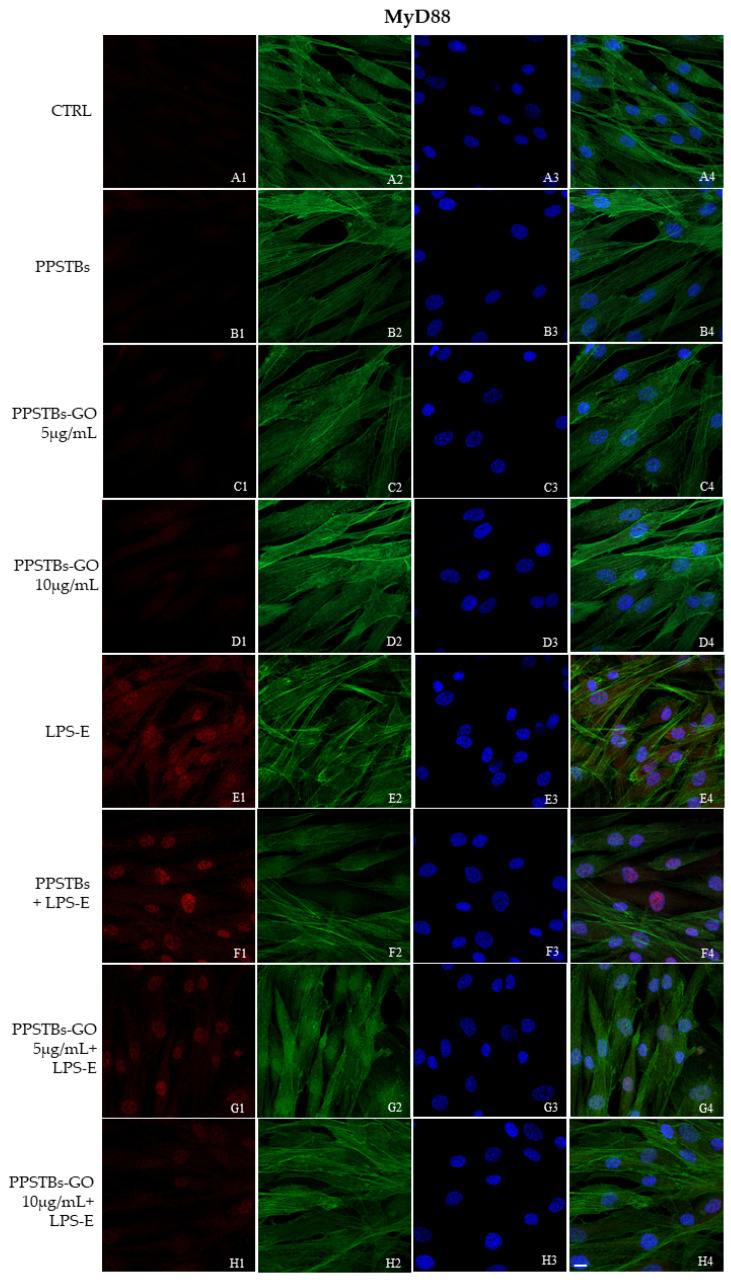
MyD88 signaling pathway in hGFs cell line. Expression of MyD88 analyzed by confocal microscopy (**A1**–**H4**), MyD88 expression untreated cells (CTRL), in hGFs cultured with PPSTBs, in hGFs cultured with PPSTBs enriched with GO at 5 μg/mL, in hGFs cultured with PPSTBs enriched with GO at 10 μg/mL, in hGFs stimulated with LPS-E, in hGFs cultured with PPSTBs and stimulated with LPS-E, in hGFs cultured with PPSTBs enriched with GO at 5 μg/mL and stimulated with LPS-E and in hGFs cultured with PPSTBs enriched with GO at 10 μg/mL and stimulated with LPS-E. Red fluorescence: Red fluorescence: MyD88 (**A1**–**H1**); Green fluorescence: cytoskeleton actin (**A2**–**H2**); Blue fluorescence: cell nuclei (**A3**–**H3**) and Merge (**A4**–**H4**). Scale bar: 20 μm.

**Figure 7:**
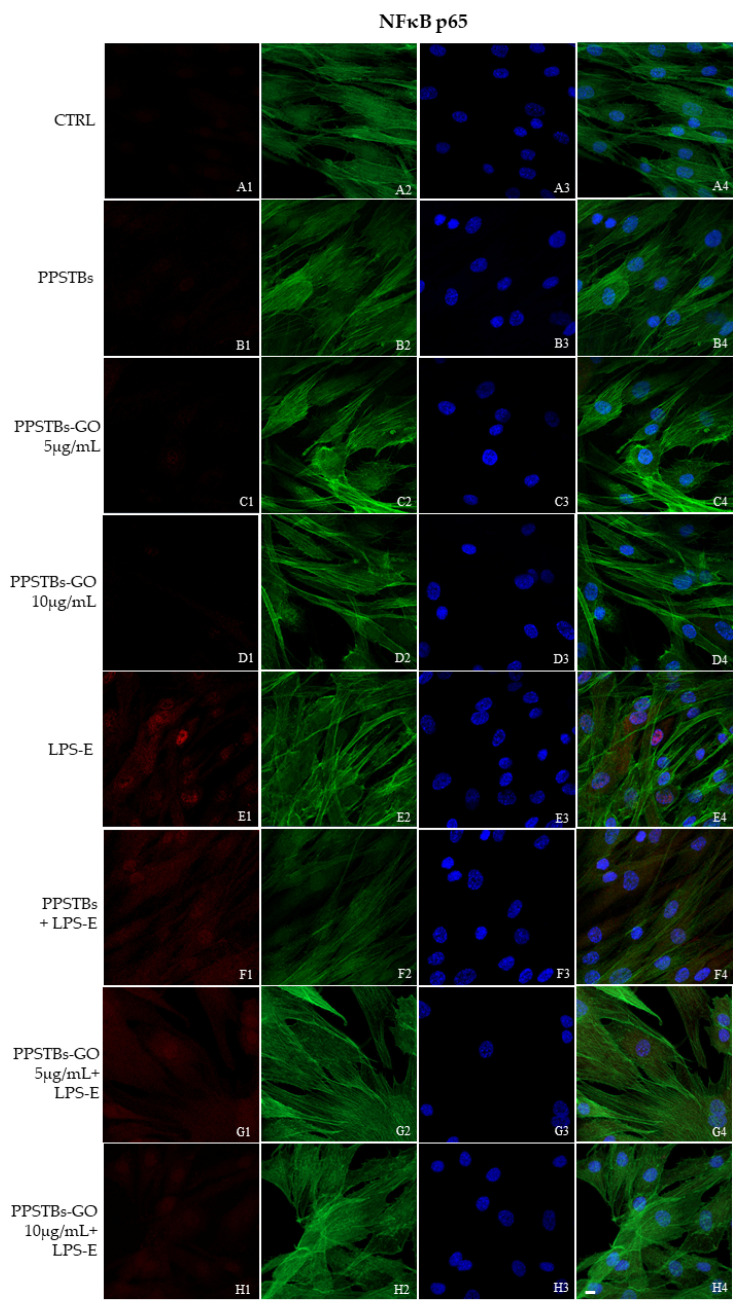
NFκB p65 signaling pathway in hGFs cell line. Expression of NFκB analyzed by confocal microscopy (**A1**–**H4**), NFκB p65 expression in untreated cells (CTRL), in hGFs cultured with PPSTBs, in hGFs cultured with PPSTBs enriched with GO at 5 μg/mL, in hGFs cultured with PPSTBs enriched with GO at 10 μg/mL, in hGFs stimulated with LPS-E, in hGFs cultured with PPSTBs and stimulated with LPS-E, in hGFs cultured with PPSTBs enriched with GO at 5 μg/mL and stimulated with LPS-E and in hGFs cultured with PPSTBs enriched with GO at 10 μg/mL and stimulated with LPS-E. Red fluorescence: Red fluorescence: NFκB p65 (**A1**–**H1**); Green fluorescence: cytoskeleton actin (**A2**–**H2**); Blue fluorescence: cell nuclei (**A3**–**H3**) and Merge (**A4**–**H4**). Scale bar: 20 μm.

**Figure 8:**
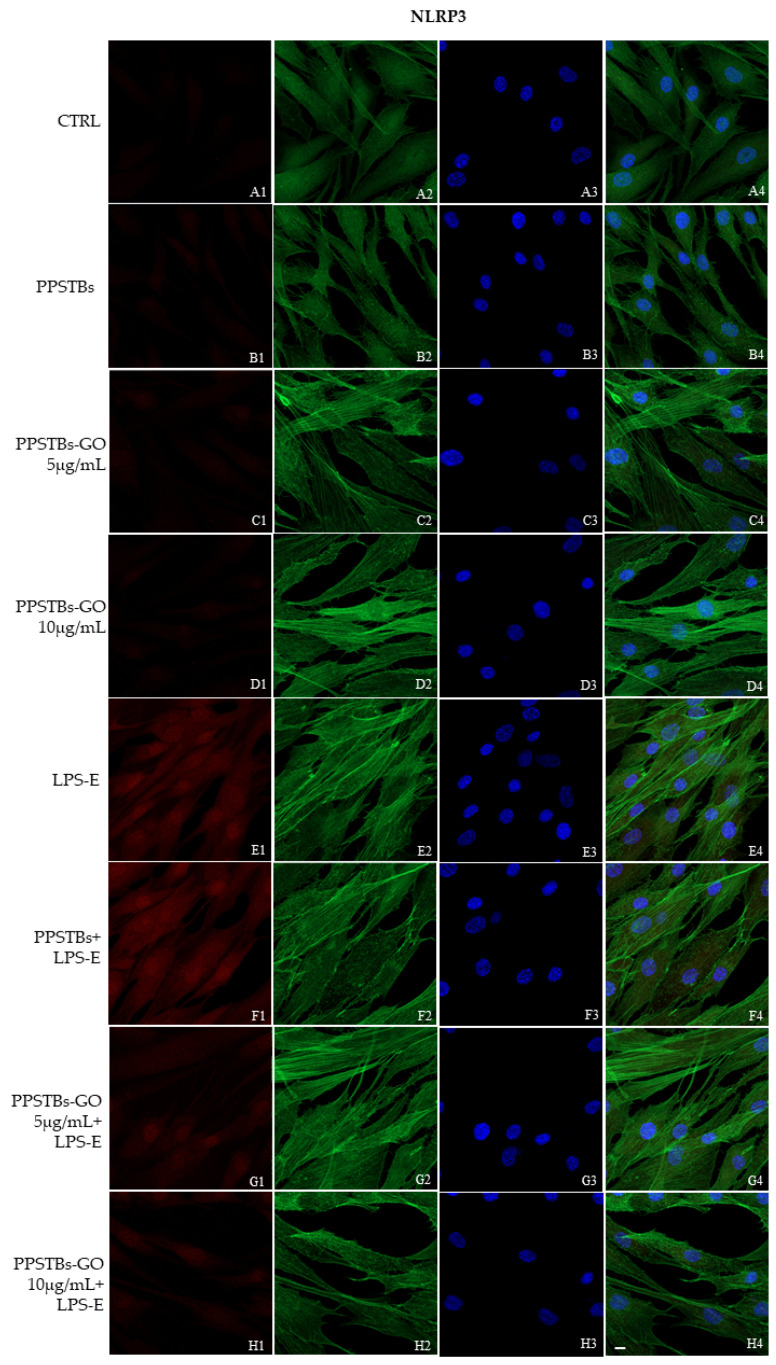
NLRP3 signaling pathway in hGFs cell line. Expression of NLR3 analyzed by confocal microscopy (**A1**–**H4**), NLRP3 expression untreated cells (CTRL), in hGFs cultured with PPSTBs, in hGFs cultured with PPSTBs enriched with GO at 5 μg/mL, in hGFs cultured with PPSTBs enriched with GO at 10 μg/mL, in hGFs stimulated with LPS-E, in hGFs cultured with PPSTBs and stimulated with LPS-E, in hGFs cultured with PPSTBs enriched with GO at 5 μg/mL and stimulated with LPS-E and in hGFs cultured with PPSTBs enriched with GO at 10 μg/mL and stimulated with LPS-E. Red fluorescence: Red fluorescence: NLRP3 (**A1**–**H1**); Green fluorescence: cytoskeleton actin (**A2**–**H2**); Blue fluorescence: cell nuclei (**A3**–**H3**) and Merge (**A4**–**H4**). Scale bar: 20 μm.

**Figure 9:**
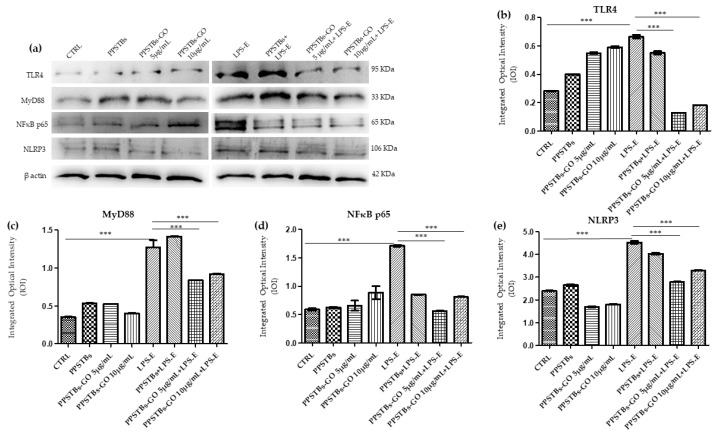
Western blotting analysis. TLR4, MyD88, NFκB p65 and NLRP3 protein expression in the untreated hGFs cells (CTRL), in hGFs cultured with PPSTBs, in hGFs cultured with PPSTBs enriched with GO at 5 μg/mL, in hGFs cultured with PPSTBs enriched with GO at 10 μg/mL, in hGFs stimulated with LPS-E, in hGFs cultured with PPSTBs and stimulated with LPS-E, in hGFs cultured with PPSTBs enriched with GO at 5 μg/mL and stimulated with LPS-E and in hGFs cultured with PPSTBs enriched with GO at 10 μg/mL and stimulated with LPS-E. (**a**) Each membrane was probed with β-actin antibody to verify the loading consistency. (**b**–**e**) Histograms represent densitometric measurements of protein bands expressed as the integrated optical intensity (IOI) mean of three separate experiments. The error bars show the standard deviation (±SD). *** *p* < 0.001.

The immunofluorescence images reported the expression levels of TLR4/MyD88/NFκB p65/NLRP3 in hGFs untreated cells, in hGFs cultured with PPSTBs, in hGFs cultured with PPSTBs enriched with GO at 5 μg/mL, in hGFs cultured with PPSTBs enriched with GO at 10 μg/mL, in hGFs stimulated with LPS-E, in hGFs cultured with PPSTBs and stimulated with LPS-E, in hGFs cultured with PPSTBs enriched with GO at 5 μg/mL and stimulated with LPS-E and in hGFs cultured with PPSTBs enriched with GO at 10 μg/mL and stimulated with LPS-E. The results showed that the TLR4/MyD88/NFκB p65/NLRP3 pathway was expressed significantly in hGFs treated with LPS-E alone or in hGFs cultured with PPSTBs and LPS-E for 24 h compared to the untreated cells. Moreover, TLR4/MyD88/NFκB p65/NLRP3 level expression was less expressed in the cells cultured with PPSTBs enriched with GO and LPS-E compared to hGFs treated with LPS-E alone or in hGFs cultured with PPSTBs and LPS-E. The hGFs cultured with PPSTBs enriched with GO at 10 μg/mL had a comparable level of expression of TLR4/MyD88/NFκB p65/NLRP3 with respect to the CTRL sample group (Figure 5, Figure 6, Figure 7 and Figure 8). The results obtained by Western blot were comparable to those obtained by confocal immunofluorescence (Figure 9).

### 2.5. Genes Expression

**Figure 10:**
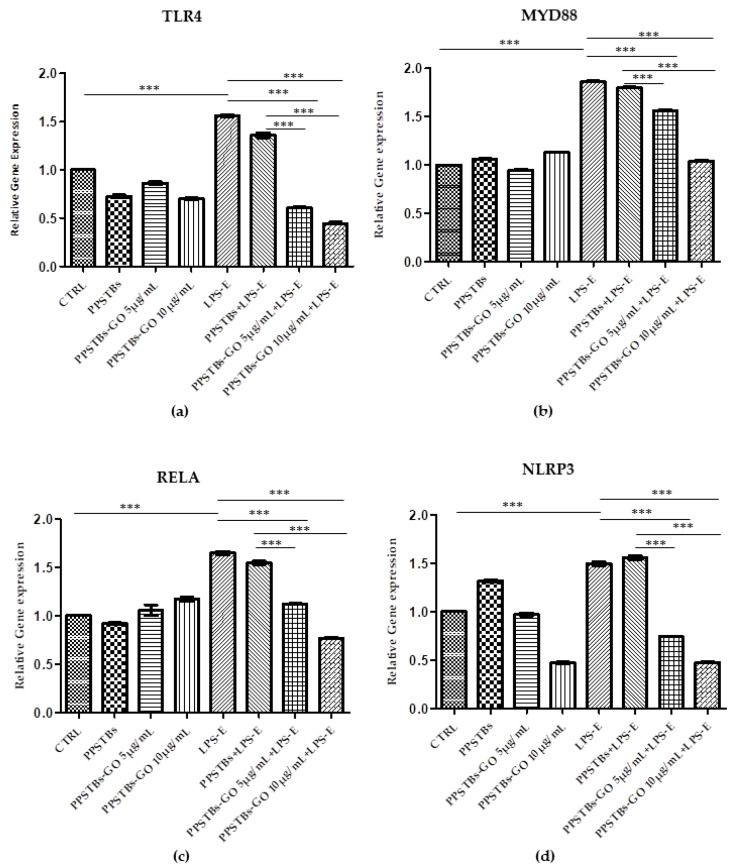
(**a**–**d**) Histograms of RT-PCR showed the mRNA levels of TLR4, MYD88, REALA, NLRP3 in untreated cells (CTRL), in hGFs cultured with PPSTBs, in hGFs cultured with PPSTBs enriched with GO at 5 μg/mL, in hGFs cultured with PPSTBs enriched with GO at 10 μg/mL, in hGFs stimulated with LPS-E, in hGFs cultured with PPSTBs and stimulated with LPS-E, in hGFs cultured with PPSTBs enriched with GO at 5 μg/mL and stimulated with LPS-E and in hGFs cultured with PPSTBs enriched with GO at 10 μg/mL and stimulated with LPS-E. *** *p* < 0.001.

**Figure 11:**
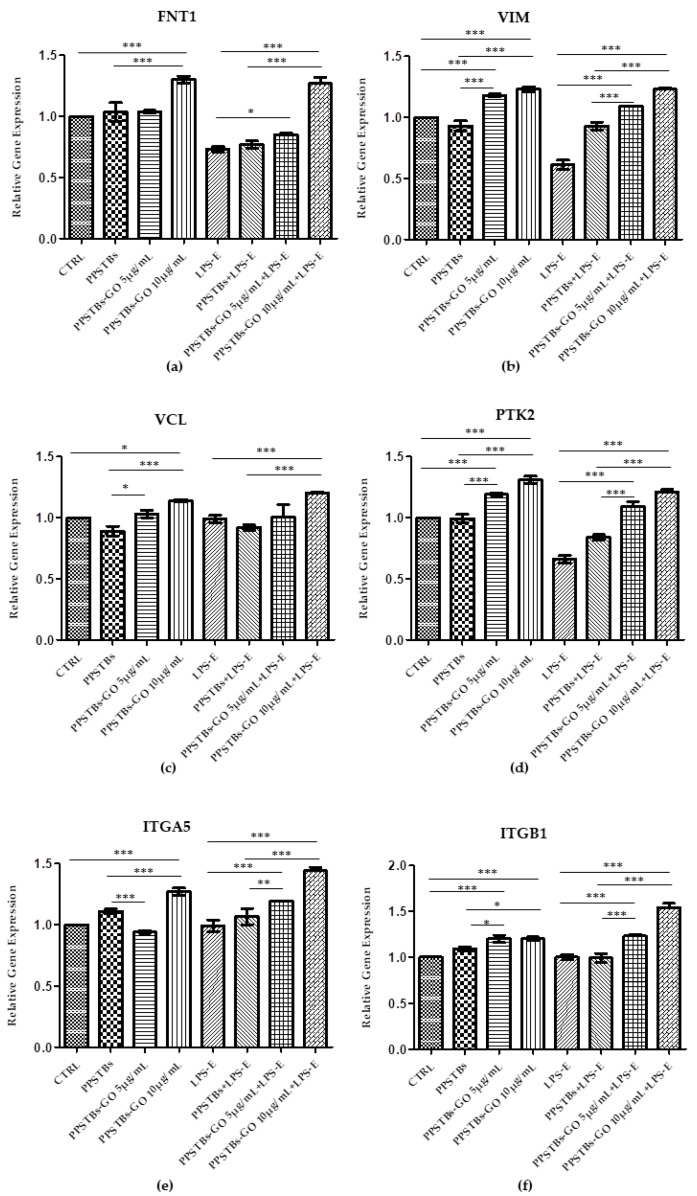
(**a**–**f**) Histograms of RT-PCR showed the mRNA levels of FNT1, VIM, VCL, PTK2, ITGA5, and ITG1b in untreated cells (CTRL), in hGFs cultured with PPSTBs, in hGFs cultured with PPSTBs enriched with GO at 5 μg/mL, in hGFs cultured with PPSTBs enriched with GO at 10 μg/mL, in hGFs stimulated with LPS-E, in hGFs cultured with PPSTBs and stimulated with LPS-E, in hGFs cultured with PPSTBs enriched with GO at 5 μg/mL and stimulated with LPS-E and in hGFs cultured with PPSTBs enriched with GO at 10 μg/mL and stimulated with LPS-E. * *p* < 0.05; ** *p* < 0.01; *** *p* < 0.001.

Histogram showed the gene expression of TLR4/MYD88/RELA/NLRP3 and FN1/VIM/VCL/PTK2/ITGA5/ITGA1 evaluated by Real-Time PCR (Figure 10 and Figure 11). The hGFs treated with LPS-E reported a significantly higher gene expression of TLR4/MYD88/RELA and NLRP3 compared to the untreated cells. Moreover, hGFs cultured with PPSTBs enriched with GO at 5 μg/mL and 10 μg/mL and stimulated with LPS-E evidenced a remarkably lower gene expression compared to hGFs stimulated with LPS-E confirming the qualitative results obtained by CLSM observations and Western blot analysis (Figure 5, Figure 6, Figure 7, Figure 8 and Figure 9). Conversely, the expression of the FNT1/VIM/VCL/PTK2/ITGA5 and ITG1b genes was significantly lower in the hGFs and PPSTBs samples compared to the PPSTBs samples enriched with both GO concentrations. The same results were shown in the samples treated with LPS-E (Figure 11a–f).

## 3. Discussion

GO plays a pivotal role in the biological and medical field, as well as in tissue repair, due to its ability to enhance cell adhesion, proliferation, and differentiation. In addition, GO possesses anti-inflammatory and antibacterial properties. As reported by Radunovic et al., many biomaterials, such as titanium disks and collagen membranes functionalized with GO, showed a reduced bacterial biofilm formation when compared with non-functionalized biomaterials [15]. AFM was used to characterize the surface morphology of the samples. AFM height images of PPSTBs-GO 5 μg/mL (Figure 1E) and PPSTBs-GO 10 μg/mL (Figure 1H) samples showed a less uniform morphology compared to the PPSTBs sample (Figure 1B). Indeed, the dispersion of GO in PP comprises the establishment of new interactions between PP and GO that require the breaking of PP intermolecular interactions. This rearrangement implies a complete reorganization of PP molecules around GO sheets and may alter the apparent morphology of the PPSTBs-GO compared to that of pure PPSTBs. Nevertheless, no relevant differences in surface roughness were observed on GO-enriched samples in comparison with PPSTBs without GO.

As far as the stiffness is concerned, the obtained Young’s elastic modulus values demonstrate that the addition of GO at a concentration of 5 μg/mL did not influence the stiffness characteristics of the starting material. On the contrary, the increase in the elastic modulus of PP composites in the presence of 10 μg/mL was well-defined, and it can be attributed to stress transfer from the polymer matrix to well-dispersed strong GO sheets, enhancing the mechanical properties of the material.

Similarly, XRD analysis does not evidence the presence of aggregated/layered GO, confirming the good dispersion of the GO in the PP matrix. The disappearance of the β phase in PPSTBs enriched GO agrees with the different mechanical properties observed by AFM, at least for the highest investigated concentration of GO [38].

In the present work, the biological effects of PPSTBs with GO in an in vitro model of hGFs were evaluated in the inflammatory process through modulation of the TLR4/MyD88/NFκB p65/NLRP3 pathway. Toll-like receptors family (TLRs) are receptors present on cell surfaces or in internal compartments such as ERs, endosomes, and lysosomes. These are formed by an ectodomain responsible for the recognition of pathogen-associated molecular patterns (PAMPs) and damage-associated molecules patterns (DAMPs) by a transmembrane domain and a cytoplasmic domain Toll/IL-1 receptor (TIR), which intervenes in the activation of downstream signaling [39]. LPS binds and activates TLR4 through the formation of a complex composed of LPS binding protein (LBP) and accessory proteins CD14 and MD2 [40]. In turn, the activated TLR4 binds myeloid differentiation factor 88 (MYD88), which activates the intracellular signaling cascade that ends with the phosphorylation of the inhibitors serine residues of the transcription regulator nuclear factor kappa B (NFκB) [41,42]. The activated form of NFκB is translocated from the cytoplasm to the nucleus, where it binds specific DNA elements and regulates the transcription of target genes resulting in increased IL-18, IL-6, IL-1β, tumor necrosis factor-α (TNF-α), and MCP-1 [43,44].

Based on the literature, the stimulation with LPS is responsible for the activation of the TLR4/NFκB pathway, which is involved in the increment of NOD-Like Receptor Protein 3 (NLRP3), a component of the NOD-like receptors that form the inflammasome complex [45,46]. The inflammasome is a group of intracellular protein complexes that assemble in response to PAMPs or DAMPs and induces the inflammatory reaction through the activation of caspase 1 [45]. Moreover, it has been demonstrated that LPS induces intracellular ROS and promotes the differentiation of M1 macrophages, which are key effector cells for the elimination of pathogens, virally infected, and cancer cells [47].

Our in vitro data suggest that both hGFs cells alone and cultured with PPSTBs show an increment in the expression of the inflammatory mediators TLR4/Myd88/NFκB p65/NLRP3 when treated with LPS-E. Instead, hGFs cells cultured with PPSTBs enriched with 5 μg/mL or 10 μg/mL of GO showed a significant reduction of TLR4/Myd88/NFκB p65/NLRP3 level expression. The reduction of these inflammatory mediators observed in hGFs cultured with PPSTBs enriched with both concentrations of GO showed that GO could be responsible for modulating the inflammatory process. The results are particularly relevant because the concentration of GO in these PPSTBs is very low (<0.1%).

Moreover, to further support the data obtained, the gene expression of the principal markers involved in cell adhesion, such as Fibronectin, Vimentin, Vinculin, Focal Adhesion Kinase (FAK), and Integrin α5β1, was also investigated.

Cell adhesion to extracellular matrix (ECM) proteins is essential for regenerative processes and for maintaining tissue homeostasis as well as for wound healing processes [48]. Cell adhesion is a fundamental biological event that defines cell and tissue morphogenesis by intervening in the modulation of cell differentiation, cycle, and survival. The adhesion proteins, the main players of this event, are membrane receptors that allow the cells to arrange themselves three-dimensionally to form the tissue and allow their interaction with the surrounding environment.

The affinity of the cells for the biomaterial substrate depends on the ECM molecules and represents a key factor for the development of the biomaterial [49]. In our study, an increase in the gene expression of the principal markers involved in cell adhesion processes was detected in hGFs untreated and treated with LPS-E and cultured with PPSTBs-GO 5 μg/mL and PPSTBs-GO 10 μg/mL. In detail, a significant increase of FNT1/VIM/VCL/PTK2/ITGA5 and ITG1b transcribing, respectively, for Fibronectin, Vimentin, Vinculin, Focal Adhesion Kinase (FAK), and Integrin α5β1 underlines that GO added to PPSTBs promotes cell-to-cell interactions and cell interactions with the surrounding environment.

Fibronectin is an ECM protein involved in cell adhesion, spreading, migration, proliferation, and apoptosis [50]. Its interaction with the heterodimeric cell surface glycoprotein regulates the mechanical anchoring and the formation of focal cell–cell and cell–material adhesion contacts [51]. Specifically, Integrin α5β1 is reported to be highly expressed in human fibroblasts, promoting their motility and survival [52,53], as well as in hGFs [54]. The interaction of Fibronectin with Integrins determines a receptor conformational change and its consequent activation resulting in a mechanical coupling to the ligand. Successively, the receptors form an adhesion complex containing structural proteins, such as Vinculin, and signaling molecules, such as FAK involved in the association with cytoskeletal actin and cell anchoring as well as in sending signals relating to ECM [55,56]. Vimentin, known as one of the principal proteins of cell intermediate filaments, is reported to enhance integrin α5β1 binding fibronectin and to improve cell–cell interactions through its association with hemidesmosomes and desmosomes [57,58].

Despite the limitations of the present in vitro study, relevant and positive outcomes have been obtained. Taken together, these findings highlight the anti-inflammatory effects and the capacity of GO to improve cell adhesion abilities, which may play an important role in the early stage of wound healing. Understanding the mechanisms of the release of ECM components and their regulation is essential for developing novel strategies in the field of tissue engineering and regenerative medicine.

The biological effects of GO, evidenced by our data, could result in better and faster healing of the tissues treated with suture thread enriched with GO. Consequently, the potential use in the clinical setting of these sutures enriched with GO could reduce hospitalization times of treated patients and limit, thank also to the demonstrated antibiofilm activity [9], the use of postoperative antibiotic therapies.

## 4. Materials and Methods

### 4.1. Graphene Oxide (GO)

**Figure 12:**
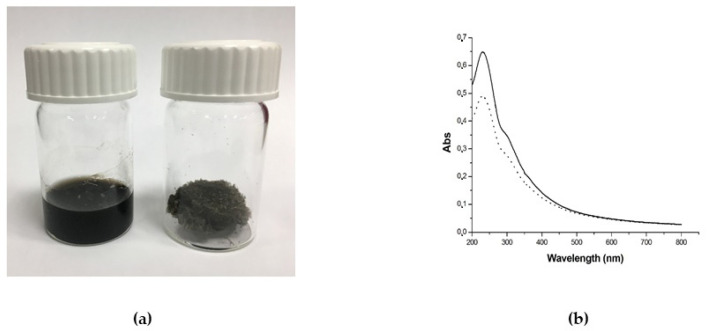
(**a**) Photograph of GO aqueous dispersion 1 mg/mL (left) and the obtained GO sponge after freeze-drying process (right) and (**b**) UV-Vis spectra of GO aqueous dispersion before freeze-drying (straight line) and GO aqueous dispersion obtained by redispersion of GO sponge (dotted line). The decrease of the absorbance after lyophilization is due to the loss of material during the process.

The GO aqueous solution was obtained as a commercial sample from Graphenea (Graphenea, Donostia-San Sebastian, Spain) and already characterized by the manufacturing company in terms of exfoliation (monolayer content > 95%), size (<10 μm), and oxidation degree (Elemental analysis: carbon: 49–56%; oxygen: 41–50%). This characterization is very important because it has been proven that the above-mentioned “biological” properties of GO depend strictly on those features. Due to the good properties of this commercial sample, we decided to use this material and add it as a solid [59]. The commercial aqueous solution of 4 g/L GO was added to Ultrapure MilliQ water (electric resistance > 18.2 MΩ cm^−1^) from a Millipore Corp. model Direct-Q 3 system (Merk, Burlington, Massachusetts, US) in order to reach the concentration of 1 mg/mL, and bath ultrasonicated for 10 min (37 kHz, 180 W; Elmasonic P60H; Elma). The concentration of GO has checked spectrophotometrically at λ_max_ 230 nm by using a Varian Cary 100 BIO UV-Vis spectrophotometer. Dimensions of GO flakes were measured by using dynamic laser light scattering (DLS) (90Plus/BI-MAS ZetaPlus multi-angle particle size analyzer; Brookhaven Instruments Corp., Holtsville, NY, US) in order to check that micrometric GO have been obtained, and ultrasonication did not reduce significantly GO flakes dimensions (see Figure S1, Supplementary Materials). In order to further characterize the commercial GO, ζ-potential measurements and Raman spectroscopy (XploRA PLUS, HORIBA, Kyoto, Japan) analyses have been performed (see Supplementary Materials). GO dispersion was divided into different aliquots and transferred at −80 °C overnight. After GO aliquots were completely frozen, the samples were placed in a freeze dryer for 48 h, generating black GO sponges. An aliquot of dried GO was redispersed in water and characterized by DLS, ζ-potential (see Supplementary Materials), and UV-Vis spectrophotometry. The UV-Vis spectrum was registered in order to observe the dispersion behavior and check the real final concentration of GO after the freeze-drying process (Figure 12). Analogously, Raman spectroscopy analyses were performed on the dry GO sample after lyophilization (see Figure S2 in the Supplementary Materials). The amount of GO necessary for the production of 50 g PPSTBs was 5 mg GO for 5 μg/mL samples and 10 mg GO for 10 μg/mL samples.

### 4.2. GO-Enriched PPSTBs

PPSTBs of pure PP were enriched with two different concentrations of GO. Briefly, 50 g of PPSTBs were dissolved at the temperature of 160 °C and thoroughly mixed with 5 mg GO for 5 μg/mL samples and 10 mg GO for 10 μg/mL samples, respectively. As the final step, the molten product was placed in molds to create the PPSTBs (produced and furnished by Assut Europe S.p.A).

### 4.3. PPSTBs, PPSTBs-GO 5 μg/mL, PPSTBs-GO 10 μg/mL Characterization

The PPSTBs substrates were characterized by AFM using the MultiMode 8 AFM microscope (Bruker, Billerica, MA, USA) equipped with a Nanoscope V controller. The Peak Force Quantitative Nanomechanics (PFQNM) mode was used to map the morphology and to acquire quantitative insight into nanomechanical parameters of PPSTBs substrates, such as Young’s elastic modulus. The PPSTBs and PPSTBs-GO 5 μg/mL samples were mapped using a precalibrated RTESPA-300-30 probe (spring constant 38.904 N/m and resonance frequency of 350.251 kHz), while for PPSTBs-GO 10 μg/mL samples, the precalibrated RTESPA-525-30 cantilever (spring constant 266.124 N/m and resonance frequency of 582.946 kHz) was chosen. The deflection sensitivity of both types of cantilevers was measured against a standard Sapphire 12-M sample, and after the calibration, images of 512 × 512 pixels were collected with scan sizes of 5 × 5 μm. To analyze the images, the Nanoscope Analysis 1.8 software was used [60]. The elastic modulus values were calculated by using the Derjaguin–Muller–Toropov (DMT) model, extracting them from each force-distance curve registered at each point of the scanned surface. XRD analysis was performed using the D2 Phaser X-ray diffractometer apparatus (Bruker, Billerica, MA, USA) with Cu Kα radiation (λ = 0.154 nm, 30 kV, 10 mA) as an X-ray source. Scattered X-ray intensities were collected over a range of scattering angle 2*ϴ* = 5° to 50° with a scan velocity of 0.05 2θ s^−1^.

### 4.4. Cell Culture

hGFs (PCS-201-018 ATCC, Manassas, VT, USA) were cultured in Fibroblast Basal Medium (ATCC PCS-201-030) in addition to Fibroblast Growth Kit-Low Serum (ATCC PCS-201-041), containing 5 ng/mL of rh FGF b, 7.5 mM of L-glutamine, 50 μg/mL Ascorbic acid, 1 μg/mL of Hydrocortisone Hemisuccinate, 5 μg/mL of rh Insulin and 2% Fetal Bovine Serum. The culture was maintained in an incubator at 37 °C in a humidified atmosphere with 5% CO_2_ and 95% air, and when the cells reached 75–80% confluence, subcultures were produced.

### 4.5. Experimental Study Design

The experimental points shown in the following study design were performed in triplicate with hGFs at passage 5. The cells were stimulated with LPS derived from *E. coli* O55:B5 (LPS-E) (L6529, Sigma-Aldrich, Milan, Italy)

- hGFs used as negative control (CTRL);
- hGFs cultured with PPSTBs for 24 h;
- hGFs cultured with PPSTBs-GO 5 μg/mL for 24 h;
- hGFs cultured with PPSTBs-GO 10 μg/mL for 24 h;
- hGFs cultured with 5 μg/mL of LPS-E for 24 h;
- hGFs cultured with PPSTBs and 5 μg/mL of LPS-E for 24 h;
- hGFs cultured with PPSTBs-GO 5 μg/mL and 5 μg/mL of LPS-E for 24 h;
- hGFs cultured with PPSTBs-GO 10 μg/mL and 5 μg/mL of LPS-E for 24 h.

### 4.6. Cell Viability Assay

The cell metabolic activity of hGFs, hGFs + PPSTBs, hGFs + PPSTBs-GO 5 μg/mL, and hGFs + PPSTBs-GO 10 μg/mL cultured with or without LPS-E was analyzed through the 3-(4,5-dimethylthiazol-2-yl)-5-(3-carboxymethoxyphenyl)-2-(4-sulfo-phenyl)-2H-tetrazolium (MTS) assay (CellTiter 96^®^ Aqueous One Solution Cell Proliferation Assay, Promega, Madison, WI, USA). hGFs of each experimental point were seeded at the density of 3.2 × 10^3^ cells/well into 96-well plates with Fibroblast Basal Medium (ATCC PCS-201-030) added with Fibroblast Growth Kit-Low Serum (ATCC PCS-201-041) for 24, 48, and 72 h at 37 °C. Then 20 μL/well of MTS dye solution was added to the culture medium, and the plates were incubated for 3 h at 37 °C. The cell viability, defined by formazan salts quantification, was evaluated through absorbance measurements at 490 nm wavelength performed using the Synergy™ HT Multi-detection microplate reader (Biotech, Winooski, VT, USA). The amount of formazan salts detected was directly proportional to the number of live cells in the plate. The MTS assay was executed in three independent experiments [61].

### 4.7. Microscope Optical Analysis

After 24 h of LPS-E treatment, the morphology of hGFs alone or cultured with PPSTBs, PPSTBs-GO 5 μg/mL, and PPSTBs-GO 10 μg/mL were observed at the inverted light microscope (Leica DMIL, Leica Microsystem) Mag: 10×.

### 4.8. Scanning Electron Microscopy (SEM)

hGFs cells were seeded on PPSTBs, PPSTBs-GO 5 μg/mL, and PPSTBs-GO 10 μg/mL attached to the bottom of a 12-well plate with and without stimulation with LPS-E. After 24 h of culture, cells were fixed for 1 h at 4 °C in 2.5% glutaraldehyde (Electron Microscopy Sciences, EMS, Hatfield, PA, USA), in 0.1 M sodium phosphate buffer (PB), pH 7.3, rinsed three times with PB, and post-fixed for 1 h in 1% aqueous osmium tetroxide (EMS) at 4 °C. The cells were dehydrated through an ethanol series (30%, 50%, 70%, 90%, 95%, and two times 100%) followed by drying in air and carbon. Morphological analysis was carried out using a high-resolution scanning electron microscope (SEM) Regulus 8220 (Hitachi, Ltd., Tokyo, Japan) operated at 1 kV.

### 4.9. Confocal Laser Scanning Microscope (CLSM)

The hGFs were cultured in 8-well culture glass slides (Corning, Glendale, AZ, USA) at the density of 1.3 × 10^4^/well. After 24 h of treatment, the cells were fixed 1 h at room temperature with 4% of paraformaldehyde (PFA) (BioOptica, Milan, Italy) in 0.1 M in PBS (Lonza, Basel, Switzerland). After 3 washes in PBS, the cells were permeabilized with 0.1% Triton X-100 (BioOptica) in PBS for 5–6 min and blocked with 5% of non-fat milk in PBS for 1 h at RT. Successively, the primary antibodies were prepared in 2.5% non-fat milk in PBS and maintained overnight at 4 °C. The primary antibody used in this study were all purchased from Santa Cruz Biotechnology (Dallas, TX, USA) and were used, as suggested by their datasheet, at the concentration of 1:200: TLR4 (sc-293072), anti-MyD88 (sc-74532), anti-NFκB p65 (sc-8008), anti-NLRP3 (sc-134306). The secondary antibody Alexa Fluor 568 red fluorescence-conjugated goat anti-mouse (A11031, Invitrogen, Eugene, OR, USA) has been prepared 1:200 in 2.5% non-fat milk in PBS and added 1 h at 37 °C. The cytoskeleton actin and the nuclei have been stained, respectively, with Alexa Fluor 488 phalloidin green fluorescent conjugate (A12379, Invitrogen) and TOPRO (T3605, Invitrogen), both prepared 1:200 in 2.5% non-fat milk in PBS and maintained 1 h at 37 °C. The images were acquired through Zeiss LSM800 confocal system (Carl Zeiss, Jena, Germany) [62].

### 4.10. Western Blotting Analysis

The lysates of hGFs (50 μg) underwent electrophoresis and were moved to a polyvinylidenfluoride (PVDF) membrane using a SEMI-dry blotting apparatus (Bio-Rad Laboratories Srl, Milan, Italy). Successively, the membranes were blocked in 5% non-fat milk in PBS 0.1% Tween-20 (Sigma-Aldrich) and then incubated overnight at 4 °C with the following primary antibodies: anti-TLR4 (1:500) (sc-293072, Santa Cruz Biotechnology), anti-MyD88 (1:500) (sc-74532, Santa Cruz Biotechnology), anti-NFκB p65 (1:500) (sc-8008, Santa Cruz Biotechnology), anti-NLRP3 (1:500) (sc-134306, Santa Cruz, Biotechnology), and β-actin as loading control (1:750) (sc-47778, Santa Cruz Biotechnology). After five washings with PBS 0.1% Tween-20, the membranes were incubated for 1 h at room temperature with peroxidase-conjugated secondary antibody goat anti-mouse (A90-116P, Bethyl Laboratories Inc., Montgomery, TX, USA) 1:5000 diluted in 2.5% no-fat milk in PBS and 0.1% Tween-20%. The expression levels of the proteins were detected using the enhanced chemiluminescence exposure process (ECL) (Amersham Pharmacia Biotech, Milan, Italy) with an image documenter Alliance 2.7 (Uvitec, Cambridge, UK). The detected signals were analyzed by ECL enhancement and assessed through UVIband-1D gel analysis (Uvitec). The data obtained were normalized with values assessed by densitometric analysis of the β-actin protein. The Western blotting analysis was executed in three independent experiments [63].

### 4.11. RNA Isolation and Real-Time RT-PCR Analysis

TLR4, MyD88, NFκB p65, and NLRP3 mRNA expression were analyzed by Real-Time PCR. Total RNA was extracted using PureLink RNA Mini Kit (Ambion, Thermo Fisher Scientific, Milan, Italy) according to the manufacturer’s instructions. Three independent biological replicates were analyzed for each sample. One microgram of total RNA was retrotranscribed using M-MLV Reverse Transcriptase (M1302 Sigma-Aldrich) to synthesize cDNA for 10 min at 70 °C, 50 min at 37 °C and 10 min at 90 °C according to the technical bulletin. Real-Time PCR was performed with Mastercycler ep real plex Real-Time PCR system (Eppendorf, Hamburg, Germany). The levels of mRNA expression of TLR4, MYD88, RELA, NLRP3, FN1, VIM, VCL, PTK2, ITGA5, ITG1B, and Beta-2 microglobulin (B2M) (endogenous marker) were evaluated in hGFs cells cultured alone, in hGFs cultured with PPSTBs, in hGFs cultured with PPSTBs enriched with GO at 5 μg/mL, in hGFs cultured with PPSTBs enriched with GO at 10 μg/mL, in hGFs stimulated with LPS-E, in hGFs cultured with PPSTBs and stimulated with LPS-E, in hGFs cultured with PPSTBs enriched with GO at 5 μg/mL and stimulated with LPS-E and in hGFs cultured with PPSTBs enriched with GO at 10 μg/mL and stimulated with LPS-E. Commercially available PrimeTime™ Predesigned qPCR Assays TLR4 (Hs.PT.58.38700156.g, Tema Ricerca Srl, Castenaso, Italy); RELA (Hs.PT.58.22880470, Tema Ricerca Srl) MYD88 (Hs.PT.58.40428647.g, Tema Ricerca Srl), NLRP3 (Hs.PT.58.39303321, Tema Ricerca Srl) FN1 (Hs.PT.58.40005963, Tema Ricerca Srl), VIM (Hs.PT.58.38906895; Tema Ricerca Srl), VCL (Hs.PT.58.2753988, Tema Ricerca Srl), PTK2 (Hs.PT.58.524947 Tema Ricerca Srl), ITGA5 (Hs.PT58.4796384 Tema Ricerca Srl), ITGB1 (Hs.PT.58.39883300 Tema Ricerca Srl) and the PrimeTime™ Gene Expression Master Mix (cat.n°1055772, Tema Ricerca Srl) were utilized according to standard protocols (Table 1). Beta-2 microglobulin (B2M Hs.PT.58v.18759587, Tema Ricerca Srl) was utilized for template normalization. The amplification program included a preincubation step for cDNA denaturation (3 min at 95 °C), followed by 40 cycles consisting of a denaturation step (15 s at 95 °C) and an annealing step (1 min at 60 °C). Expression levels for each gene were performed according to the 2^−ΔΔCt^ method. Real-Time PCR was performed in three independent experiments.

### 4.12. Statistical Analysis

Statistical significance was established with GraphPad 5 (GraphPad, San Diego, CA, USA) software utilizing one-way ANOVA followed by post hoc Tukey’s multiple comparisons analysis. Values of *p* < 0.05 were considered statistically significant.

## 5. Conclusions

The current work aimed to investigate the possible therapeutic benefit of commercial PP suture threads enriched with GO in a gingival fibroblasts cellular model. Our results showed that GO-fabricated PP suture threads modulated the inflammatory effects induced by LPS-E through TLR4/MyD88/NFκB p65/NLRP3 pathway. The biological effects of suture thread enriched with GO may represent a promising strategy that can be applied in clinical medicine.

## Figures and Tables

**Table 1:** Primer sequences used for real-time PCR reactions.

Gene	Forward Primer Sequence (5′-3′)	Reverse Primer Sequence (5′-3′)
TLR4	5′-GAGTATACATTGCTGTTTCCTGTTG-3′	5′-ACCCCATTAAT- TCCAGACACA-3′
MYD88	5′-CGGTCTCCTCCA- CATCCT-3′	5′-GCCGGACCCAA-GTACTCA-3′
RELA	5′-CGAGCTTGTAGGAAAGGACTG-3′	5′-TGACTGATAGC- CTGCTCCAG-3′
NLRP3	5′-GAATGCCTTGG- GAGACTCAG-3′	5′-AGATTCTGATT- AGTGCTGAGTACC-3′
FN1	5′-CGTCCTAAAGA-CTCCATGATCTG-3′	5′-ACCAATCTTGT- AGGACTGACC-3′
VIM	5′-CAAGACCTGCT- CAATGTTAAGATG-3′	5′-GTGAATCCAGA- TTAGTTTCCCTCA-3′
VCL	5′-CGATACCACAA-CTCCCATCAAG-3′	5′-AGCACCAAGCT- TTCCTGAA-3′
PTK2	5′-AACAGTGAAGACAAGGACAGAA-3′	5′-TCAGCCATATT- CTCCGCAAT-3′
ITGA5	5′-ACCAACAAGAG-AGCCAAAGTC-3′	5′-TTGTACACAGC- CTCACACTG-3′
ITG1B	5′-GTAGCAAAGGA- ACAGCAGAGA-3′	5′-GGTCAATGGGATAGTCTTCAGC-3′
B2M	5′-GGACTGGTCTT-TCTATCTCTTGT-3′	5′-ACCTCCATGAT- GCTGCTTAC-3′

## Data Availability

Data are available to the corresponding author upon request.

## Supplementary Materials

The following supporting information can be downloaded at: https://www.mdpi.com/article/10.3390/ijms24076622/s1. References [64,65,66] are cited here.
