# Supplementary material for: Enriched Graphene Oxide-Polypropylene Suture Threads Buttons Modulate the Inflammatory Pathway Induced by Escherichia coli Lipopolysaccharide
Source: Int J Mol Sci. 2023 Apr 1;24(7):6622. doi: 10.3390/ijms24076622 (PMC10095426; doi:10.3390/ijms24076622)
Supplement: Supplementary file 1 [file ijms-24-06622-s001.zip › ijms-2271857-supplementary.pdf]

International Journal of *Molecular Sciences*

MDPI

Supplementary Materials

Enriched Graphene Oxide-Polypropylene Suture Threads Buttons Modulate the Inflammatory Pathway Induced by Escherichia Coli Lipopolysaccharide

Luigia Fonticoli[1], Francesca Diomede[1,2], Antonio Nanci[3,4], Antonella Fontana [2,5], Ylenia Della Rocca[1], Dainelys Guadarrama Bello[3], Serena Pilato[5], Oriana Trubiani[1,2*], Jacopo Pizzicannella[2,6†], Guya Diletta Marconi[1,2†]

[1] Department of Innovative Technologies in Medicine & Dentistry, University "G. d'Annunzio" Chieti-Pescara, via dei Vestini, 31, 66100 Chieti, Italy
[2] UdA TechLab, University "G. d'Annunzio" Chieti-Pescara, 66100 Chieti
[3] Laboratory for the Study of Calcified Tissues and Biomaterials, Department of Stomatology, Faculty of Dental Medicine, Université de Montréal, Montréal, Québec H3C3J7, Canada
[4] Department of Biochemistry and Molecular Medicine, Faculty of Medicine, Université de Montréal, Montréal, Québec, H3C3J7, Canada
[5] Department of Pharmacy, University "G. d'Annunzio" Chieti-Pescara, Via dei Vestini, 31, 66100 Chieti, CH, Italy
[6] Department of Engineering and Geology, University "G. d' Annunzio" Chieti-Pescara, Viale Pindaro, 42, 65127 Pescara, Italy
* Correspondence: oriana.trubiani@unich.it
† These authors contribute equally to the paper as senior authors.

Citation: Fonticoli, L.; Diomede, F.; Nanci, A.; Fontana, A.; Della Rocca, Y.; Guadarrama Bello, D.; Pilato, S.; Trubiani, O.; Pizzicannella, J.; Marconi, G.D. Enriched Graphene Oxide-Polypropylene Suture Threads Buttons Modulate the Inflammatory Pathway Induced by *Escherichia Coli* Lipopolysaccharide. *Int. J. Mol. Sci.* **2023**, *24*, 6622. https://doi.org/10.3390/ijms24076622

Academic Editor: Ning Wang

Received: 23 February 2023
Revised: 29 March 2023
Accepted: 30 March 2023
Published: 1 April 2023

Copyright: © 2023 by the authors. Submitted for possible open access publication under the terms and conditions of the Creative Commons Attribution (CC BY) license (https://creativecommons.org/licenses/by/4.0/).

1. Characterization of commercial GO

Dynamic laser light scattering (DLS), ζ-potential, and Raman spectroscopy measurements were performed in order to confirm the micrometric dimensions and the exfoliation degree of GO flakes before and after the sample underwent lyophilization.

1.1. Dynamic laser light scattering and ζ-potential measurements

Dimensions of GO flakes were measured on the commercial GO sample after dilution and sonication as well as on the aqueous solution obtained by rehydration of the lyophilized sample by using a 90Plus/BI-MAS ZetaPlus multi-angle particle size analyzer (Brookhaven Instruments Corp.). A mean value of diameter of 717.7 ± 62.0 nm was obtained for the former sample and a value of 1255.8 ± 134.2 nm for the latter sample. Figure S1 reports the dimensions of GO in aqueous solution before and after the lyophilization confirming the micrometric GO dimensions and highlighting that ultrasonication and lyophilization did not alter significantly GO flakes dimensions, considering the fact that both the samples are quite heterogeneous and polydispersed.

The values of ζ-potential of diluted GO commercial aqueous dispersion and that of GO aqueous dispersion obtained by rehydration of the lyophilized sample were -26.3±2.8 mV and -28.8±0.9 mV, respectively, indicative of the presence of electronegative oxygenated functional groups at the surface and the dissociation of carboxylic acid moieties at the edges of the GO flakes in both samples. These values are representative [1] of dispersions in which charged GO flakes are considered to possess a sufficient mutual repulsion force with each other to form a stable and well-exfoliated aqueous dispersion.

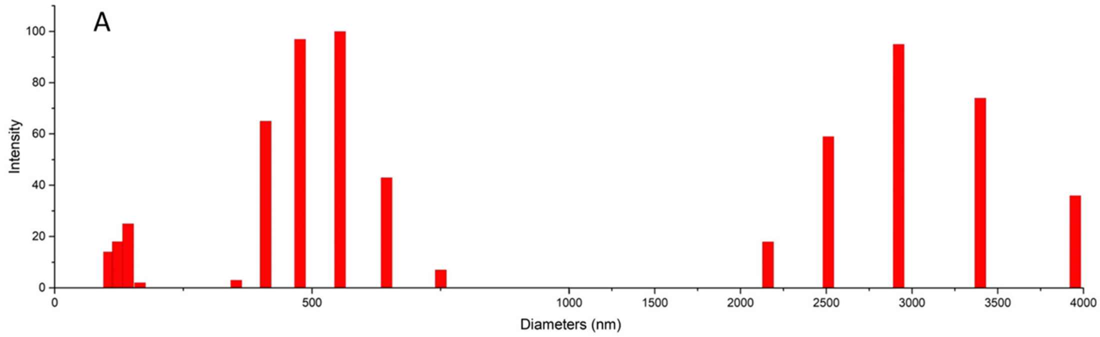

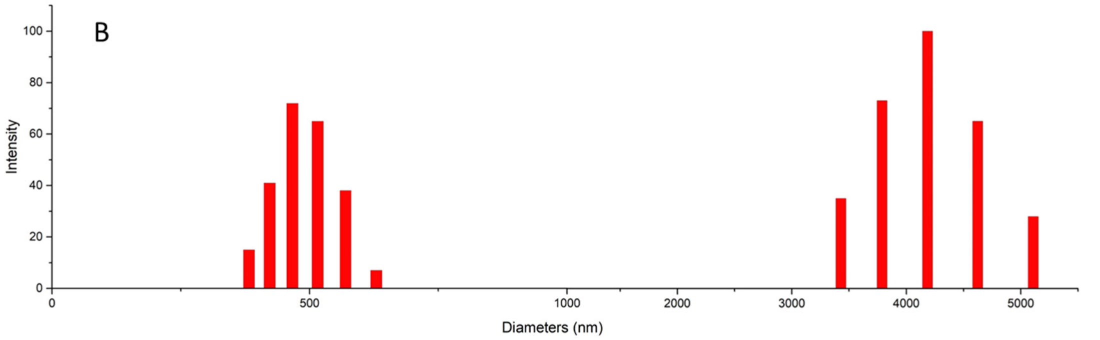

Figure S1. DLS measurement of commercial GO aqueous dispersions (A) before and (B) after lyophilization.

1.2. Raman spectroscopy analysis

In order to confirm that GO flakes were not altered during lyophilization, Raman spectra of the diluted aqueous GO solution drop-casted on the SiO_2 substrate as well as of the lyophilized sample deposited onto the SiO_2 substrate were performed.

The Raman spectra were obtained by confocal and high-performance Raman microscope (XploRA PLUS, HORIBA, Japan) in the range of 1200-3300 cm^{-1} and with an 1800-line/mm grating. The samples were detected with a 532 nm laser, with a time of 20 s and 30 accumulations. Power irradiation of not more than 10% at the sample surface was used in order to avoid laser-induced heating. LabSpec (Horiba, Japan) was employed to control the Raman spectroscopic system and to optimize and process the acquired data. Figure S2 reports the above-mentioned Raman spectra evidencing no significant difference between the two samples. The spectra of GO show both the G and the D bands, representing the planar configuration of sp^2-bonded carbons that constitute the graphene surface and the presence of defects in the graphenic structure, respectively. In particular, the G band is shifted towards a higher wavenumber (≈1600 cm^{-1} *vs.* 1570 cm^{-1}) with respect to graphene due to the oxidation of GO and the good exfoliation of the sample,[2] whereas the D band has a higher intensity with respect to graphene due to defects and disorders such as grain boundaries, aliphatic carbons, etc. present in GO [3]. The absence of the 2D band, which appears generally at 2700 cm^{-1} in graphene, is due to the breaking of the stacking order due to the oxidation reaction [3].

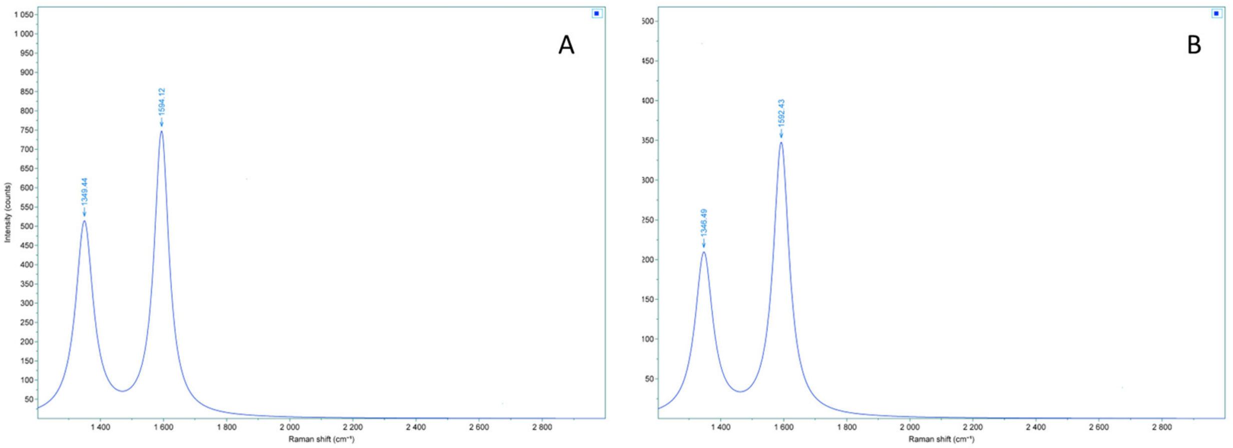

Figure S2. Raman spectra of (A) drop casted commercial GO aqueous dispersion on SiO_2 substrate and (B) lyophilized GO deposited onto SiO_2 substrate.

2. XRD measurements

XRD diffraction patterns performed on PPSTBs and PPSTBs-GO 5 and 10μg/mL are reported in Figure S3.

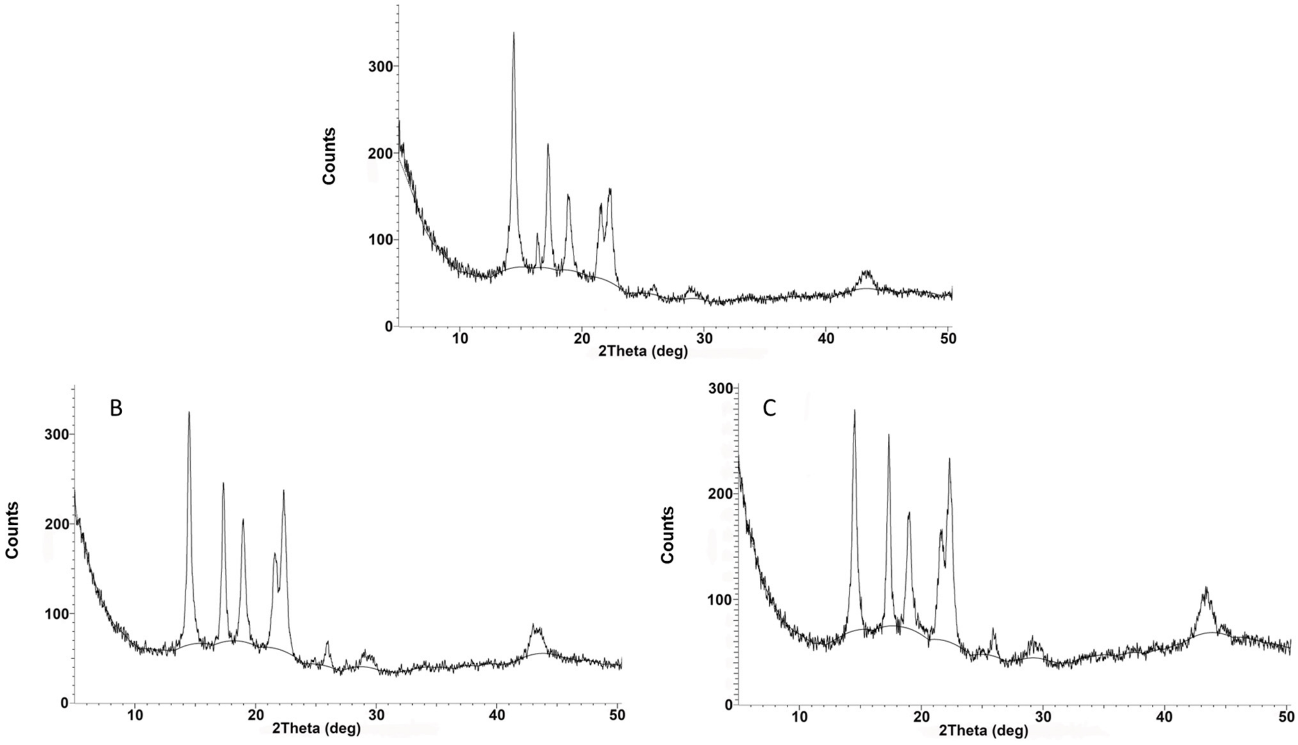

Figure S3. XRD diffraction pattern of (A) PPSTBs and (B) PPSTBs-GO 5μg/mL and (C) PPSTBs-GO 10μg/mL.

References

1. Konkena, B.; Vasudevan, S. Understanding aqueous dispersibility of graphene oxide and reduced graphene oxide through pK(a) measurements. *J. Phys. Chem. Lett.* **2012**, 3, 867-872, doi: 10.1021/jz300236w.
2. Wang, S.; Wang, R.; Liu, X.; Wang, X.; Zhang, D.; Guo, Y. Optical Spectroscopy Investigation of the Structural and Electrical Evolution of Controllably Oxidized Graphene by a Solution Method. *J. Phys. Chem. C* **2012**, 116, 10702–10707, doi: 10.1021/jp212184n.
3. Krishnamoorthy, K.; Veerapandian, M.; Yun, K.; Kim, S.-J. The chemical and structural analysis of graphene oxide with different degrees of oxidation. *Carbon* **2013**, 53, 38–49, doi: 10.1016/j.carbon.2012.10.013.

Disclaimer/Publisher's Note: The statements, opinions and data contained in all publications are solely those of the individual author(s) and contributor(s) and not of MDPI and/or the editor(s). MDPI and/or the editor(s) disclaim responsibility for any injury to people or property resulting from any ideas, methods, instructions or products referred to in the content.
